# Long-term functional outcome after conservative management of a fingertip amputation with exposed bone, using a semi-occlusive dressing: a case report

**DOI:** 10.1097/RC9.0000000000000871

**Published:** 2026-08-24

**Authors:** Eladio Collado-Boira, Luis Aguilella-Fernández, Pablo Salas-Medina

**Affiliations:** a Faculty of Health Sciences, Predepartmental Nursing Unit, Universitat Jaume I, Castelló de la Plana, Spain; b Department of Orthopaedic and Trauma Surgery, Hospital de la Ribera, Alcira, Spain

**Keywords:** case report, conservative management, exposed bone, fingertip amputation, QuickDASH, semi-occlusive dressing

## Abstract

**Introduction and importance:**

Semi-occlusive dressings are established for selected fingertip amputations with exposed bone. Because this treatment is well described, the value of single-case reports depends on documenting the course, limitations, and long-term sequelae.

**Case presentation:**

A 47-year-old right-hand-dominant man presented to primary care after a mandoline-slicer injury causing partial amputation of the right little fingertip, with an exposed distal phalanx and a preserved nail bed, clinically consistent with an Allen type II injury. A transparent, semi-occlusive polyurethane dressing was applied and changed weekly. No systemic antibiotics were prescribed.

**Clinical discussion:**

The exposed bone was clinically covered by week 4, and near-complete pulp reconstruction was observed by week 6. At a 6-year in-person follow-up, following telephone contact, the patient reported no sick leave, pain 0/10, no cold intolerance, a complete active range of motion, and no secondary surgery, infection, or clinical evidence of osteomyelitis. The QuickDASH score was 2.3/100. Sequelae included a mild reduction in temperature and pressure perception, minor typing adaptation, and a subtle lateral nail-growth alteration of approximately 2 mm.

**Conclusion:**

This single selected case documents a favorable 6-year outcome after conservative management. No general conclusion regarding treatment effectiveness can be drawn from a single case.

## Graphical Abstract

**Figure:**
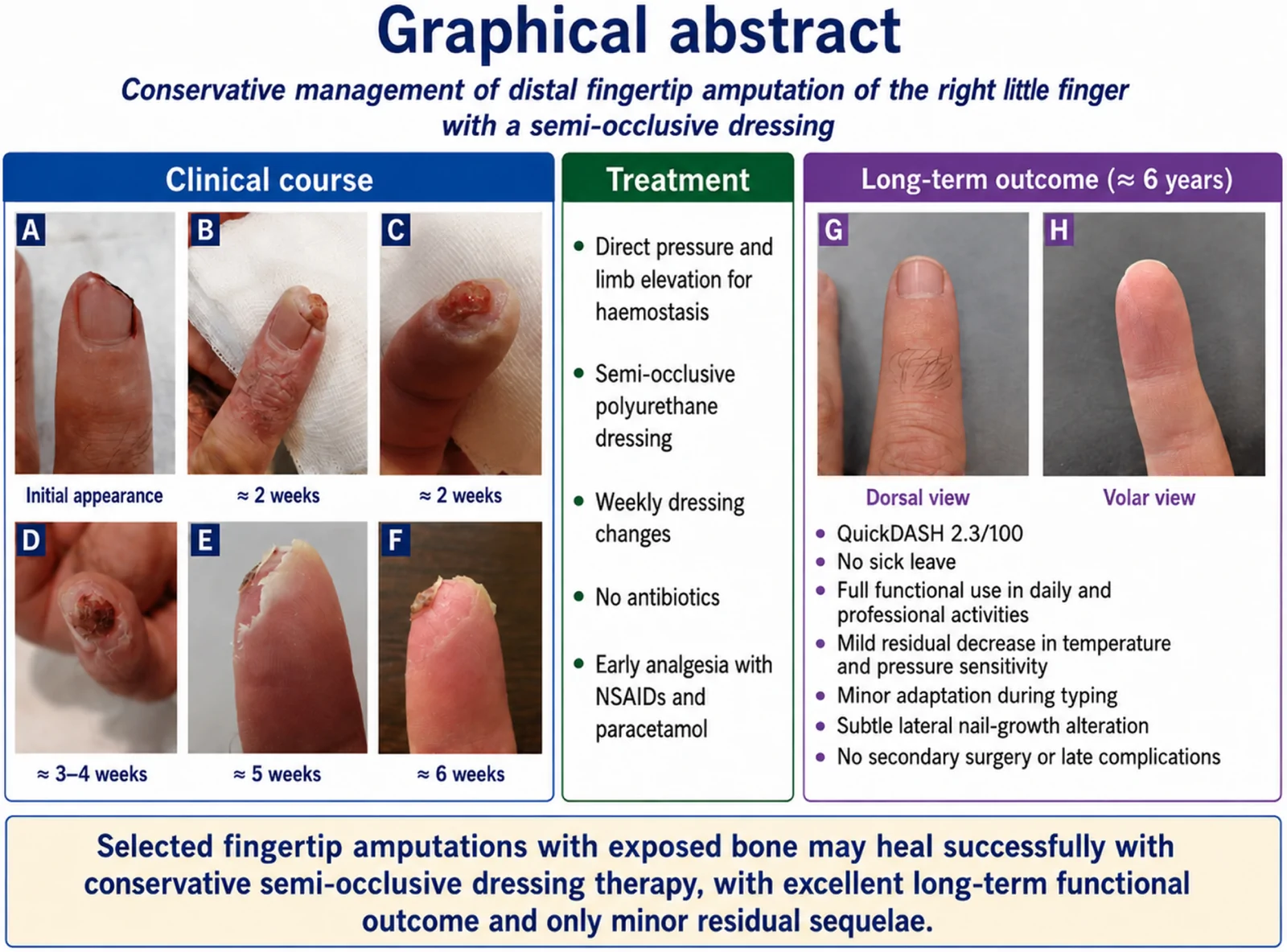

## Introduction

Distal fingertip amputations may affect sensitivity, fine motor function, appearance, work activity, and quality of life. Surgical options include closure, grafting, bone shortening, revision amputation, or flap reconstruction^[^1,2^]^. Semi-occlusive dressings support granulation, epithelialization, and soft-tissue regeneration^[^3–7^]^. This report does not present a new technique or evidence of general effectiveness. Its educational value lies in documenting one selected exposed-bone fingertip amputation managed in primary care, with chronological wound evolution, clinical bone coverage, six-year in-person follow-up, patient-reported function, minor sequelae, and uninterrupted work activity.

HIGHLIGHTS

- A distal fingertip amputation with exposed bone was treated conservatively with a semi-occlusive dressing.
- Complete bone coverage occurred by week 4, and near-complete pulp reconstruction occurred by week 6.
- Six-year follow-up showed minimal disability, with a QuickDASH score of 2.3 out of 100.
- Mild sensory- and nail-related sequelae persisted but did not limit daily or professional activities.

### Methods and reporting guidelines

This case report has been reported in line with the SCARE 2025 checklist^[^8^]^. Written informed consent was obtained for the publication of clinical details and images.

## Case presentation

On 23 April 2020, a 47-year-old man with no relevant medical history presented to a primary care center after cutting vegetables with a mandoline slicer. He was right-hand dominant and worked as a university professor. Examination showed partial amputation of the right little-finger pulp, approximately 10 × 15 mm, with an exposed distal phalanx and no nail-bed involvement (Fig. 1A). The injury was considered clinically consistent with an Allen type II injury, because the distal pulp was involved and the nail bed was preserved. The initial vascular status was preserved. Active motion was preserved, with clinically intact flexor and extensor tendon function. No gross neurological deficit was identified, although standardized sensory testing was not performed initially. There were no signs of proximal trauma, joint involvement, a foreign body, or infection.

**Figure 1.**
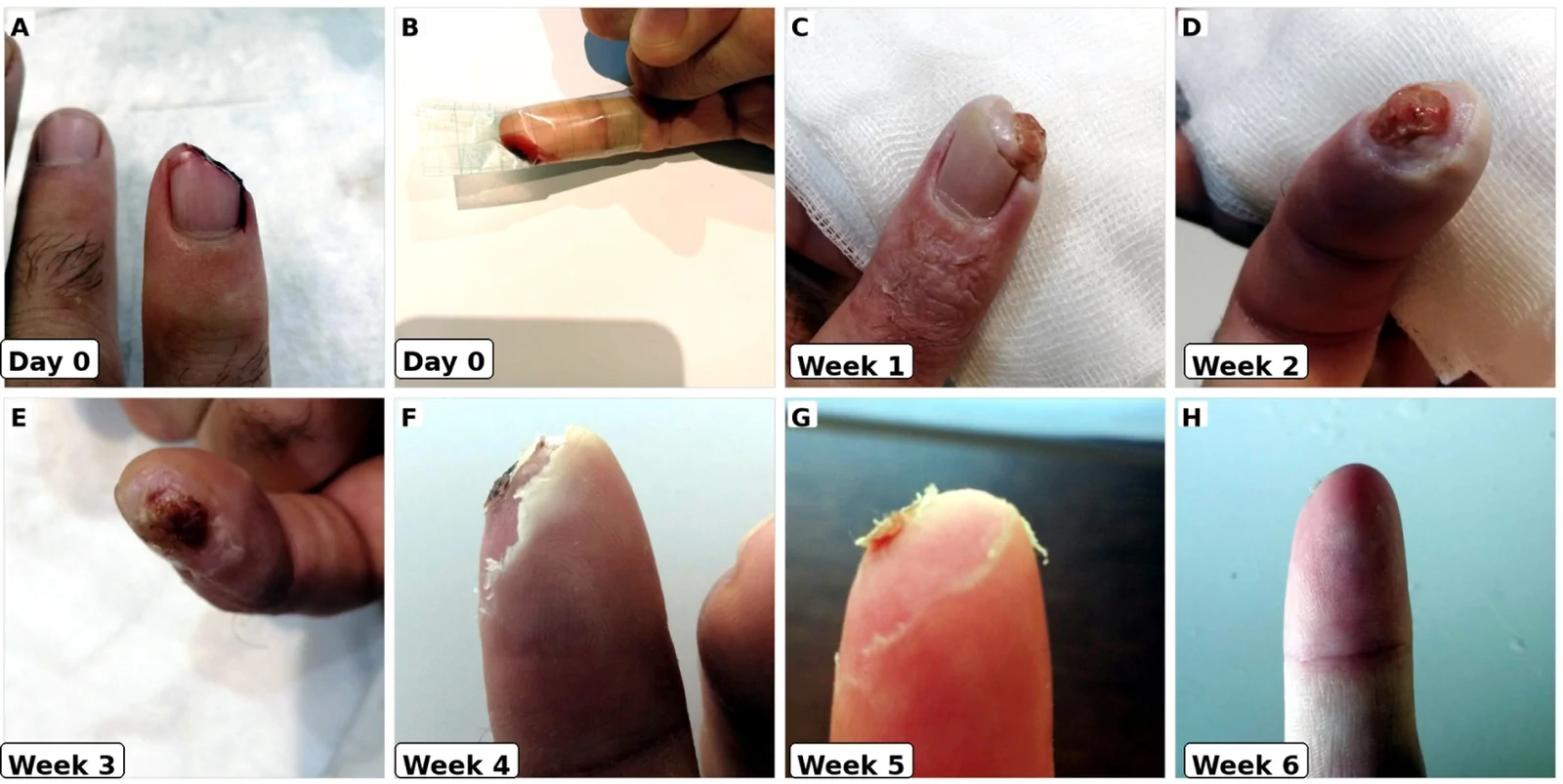
Sequential clinical evolution of the injured right little finger, managed conservatively with a semi-occlusive dressing. (A) Initial presentation (day 0), showing distal pulp amputation of the right little finger with exposed distal phalanx and preserved nail bed. (B) Application of a transparent semi-occlusive polyurethane dressing on the day of injury (day 0). (C) One-week follow-up, showing early granulation tissue formation. (D) Two-week follow-up, showing progressive granulation tissue. (E) Three-week follow-up, showing continued soft-tissue regeneration. (F) Four-week follow-up, showing clinical coverage of the previously exposed bone. (G) Five-week follow-up, showing near-complete pulp reconstruction. (H) Six-week follow-up, showing further healing with restoration of fingertip contour.

Diagnosis was clinical. Plain radiography was not performed initially; therefore, an associated distal phalanx fracture cannot be formally excluded. The decision not to perform imaging was based on the distal injury location, the absence of deformity suggesting a more proximal injury, the absence of joint involvement, preservation of active motion, and close clinical follow-up. This is acknowledged as a limitation. The diagnosis was a right little-fingertip amputation with exposed distal phalanx, clinically consistent with an Allen type II injury.

Bleeding was controlled with direct pressure and limb elevation. Replantation was ruled out because of the distal level and tissue-fragment characteristics. Conservative treatment was chosen after discussion of surgical and conservative options. The wound was cleansed with sterile saline; no bone shortening was performed and only superficial non-viable tissue was removed. A transparent semi-occlusive polyurethane dressing (Tegaderm) was applied (Fig. 1B). The patient was advised to keep the dressing dry, return weekly, and seek earlier review for increasing pain, fever, spreading erythema, purulent discharge, or other signs of infection. Tetanus status was checked. Antibiotics were not prescribed because there was no evidence of infection. Initial pain was controlled with non-steroidal anti-inflammatory drugs and paracetamol. At each dressing change, the dressing was removed, the wound inspected, and the area gently cleansed with sterile saline. No topical agent was used. Mild maceration was managed by cleansing, drying periwound skin, and reapplying the dressing.

At 1 week, malodorous serous fluid was present beneath the dressing, with early granulation but no increasing pain, fever, spreading erythema, or purulent discharge (Fig. 1C). Weekly dressing changes continued. Granulation tissue was abundant after 2 weeks (Fig. 1D); the exposed bone was clinically covered by weeks 3 to 4 (Fig. 1E and F); and near-complete pulp reconstruction was present by weeks 5 to 6 (Fig. 1G and H). At three months, the patient reported preserved function, high esthetic satisfaction, and mild residual sensory alteration.

### Long-term follow-up

Approximately 6 years after injury, the patient was contacted by telephone and scheduled for an in-person clinic assessment. He reported no sick leave and uninterrupted work. No ongoing analgesia was required after the initial days. Pain was 0/10 at rest and during usual activities. No cold intolerance was reported. Active range of motion of the right little finger was complete and comparable to the contralateral little finger. Flexor and extensor tendon function were clinically preserved. Vascular status was normal on inspection. The QuickDASH score was 2.3/100^[^9^]^. The only functional adaptation was avoiding use of the injured right little finger during keyboard typing because of mild residual sensory reduction. Mild reduction in temperature and pressure perception persisted. There was no relevant pain, hypersensitivity requiring treatment, recurrent wound problems, infection, clinical evidence of osteomyelitis, neuroma-like pain, or secondary surgery. A subtle lateral nail-growth alteration of approximately 2 mm was observed without functional impact (Fig. 2A and B).

**Figure 2.**
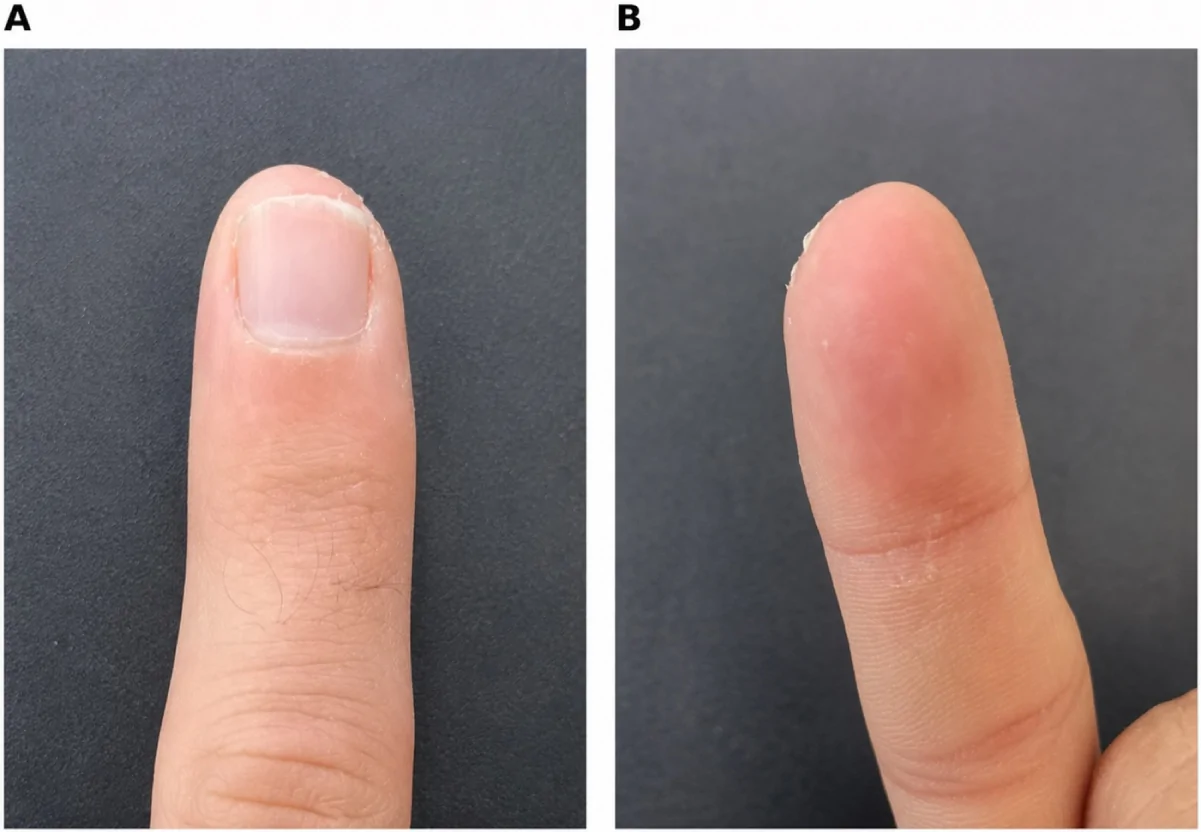
Six-year follow-up of the injured right little finger. (A) Dorsal view showing preserved overall appearance and a subtle lateral nail-growth alteration of approximately 2 mm. (B) Volar view showing preserved fingertip pulp contour without any relevant deformity.

### Patient perspective

The patient valued avoiding surgery, not requiring sick leave, and continuing normal professional activity. Mild sensory reduction and subtle nail changes were not perceived as limiting.

## Discussion

This case should be interpreted within the established literature rather than as a novel technique. Allen described conservative management, Mennen and Wiese reported semi-occlusive dressing therapy, Hoigne *et al* documented soft-tissue regeneration with exposed bone, Krauss and Lalonde reviewed secondary healing, and Pastor *et al* compared semi-occlusive dressing therapy with surgery^[^1,3–5,7^]^. The contribution is narrower: one selected primary-care case with chronological images, clinical evidence of bone coverage, an in-person 6-year follow-up, preserved professional activity, residual symptoms, and minor nail sequelae.

The clinical course matched the rationale of semi-occlusion: a protected moist environment with progressive granulation, clinical bone coverage by week 4, and near-complete pulp reconstruction by weeks 5 to 6. Malodorous serous fluid did not indicate infection because it was not accompanied by increased pain, erythema, purulence, or fever. At approximately 6 years, the patient had a QuickDASH score of 2.3/100, no sick leave, pain 0/10, no cold intolerance, a complete active range of motion, no secondary surgery, and no clinical evidence of osteomyelitis. Minor sensory and nail-related sequelae persisted. This single case cannot support conclusions regarding comparative effectiveness, costs, resource use, or surgical morbidity. Specialist assessment remains necessary for major tissue loss, an exposed tendon or joint, severe contamination, an unstable injury, uncontrolled pain, progressive infection, failure of granulation, or when patient preference or occupational demands favor reconstruction.

### Strengths and limitations

Strengths include chronological photographs, a six-year in-person follow-up, the patient perspective, a contralateral comparison, and a validated patient-reported outcome. Limitations include a single-case design, absence of initial radiography, lack of dynamometry, absence of two-point discrimination and Semmes-Weinstein testing, and limited QuickDASH sensitivity to subtle disability after a small, isolated little-finger injury.

## Conclusion

This single, selected case documents a favorable six-year outcome after conservative management of a distal fingertip amputation with exposed bone, using a semi-occlusive dressing. Its educational value lies in chronological wound documentation, clinical evidence of bone coverage, in-person follow-up, preserved function, and minor residual sensory and nail-related sequelae. No general conclusion regarding treatment effectiveness can be drawn from a single case.

## Data Availability

All relevant data are included in the article. Additional anonymized information may be available from the corresponding author on reasonable request.
